# Analysis of the Influence of Foggy Weather Environment on the Detection Effect of Machine Vision Obstacles

**DOI:** 10.3390/s20020349

**Published:** 2020-01-08

**Authors:** Zhaohui Liu, Yongjiang He, Chao Wang, Runze Song

**Affiliations:** Department of Transportation Engineering, College of Transportation, Shandong University of Science and Technology, Qingdao 266590, China; heyongjiang1213@163.com (Y.H.); chaowang@sdust.edu.cn (C.W.); songrunze0623@163.com (R.S.)

**Keywords:** faster R-CNN, foggy environment, intelligent vehicles, machine vision, object recognition

## Abstract

This study is to analyze the influence of visibility in a foggy weather environment on the accuracy of machine vision obstacle detection in assisted driving. We present a foggy day imaging model and analyze the image characteristics, then we set up the faster region convolutional neural network (Faster R-CNN) as the basic network for target detection in the simulation experiment and use Karlsruhe Institute of Technology and Toyota Technological Institute (KITTI) data for network detection and classification training. PreScan software is used to build weather and traffic scenes based on a foggy imaging model, and we study object detection of machine vision in four types of weather condition—clear (no fog), light fog, medium fog, and heavy fog—by simulation experiment. The experimental results show that the detection recall is 91.55%, 85.21%, 72.54~64.79%, and 57.75% respectively in no fog, light fog, medium fog, and heavy fog environments. Then we used real scenes in medium fog and heavy fog environment to verify the simulation experiment. Through this study, we can determine the influence of bad weather on the detection results of machine vision, and hence we can improve the safety of assisted driving through further research.

## 1. Introduction

Obstacle detection is an important guarantee of auxiliary driving safety [1], and machine vision as an important means of vehicle detection has always been of concern. In recent years, the convolutional neural network (CNN) has become a research hotspot in the field of computer vision due to its advantages in image processing tasks, and has been widely used in the field of assisted driving.

In 2012, the Alex-Net convolutional neural network designed by Alex [2] won the first place in the ImageNet image classification competition with an accuracy rate exceeding the second place of 10.9%, from then on establishing the dominant position of CNN in machine vision. In 2014, Visual Geometry Group (VGG) of Oxford University designed VGG-Nets [3]. It inherited the simple and effective characteristics of Alex-Net, and increased networks depth through convolution kernel decomposition, and improved networks efficiency by reducing parameters. However, the training process of the network becomes increasingly difficult with the deepening of the network, and the emergence of ResNet [4] solves this difficulty. Through residual function and identity mapping, ResNet can fit any target mapping and solve the gradient “diffusion” or “explosion” problem caused by too many layers. VGG-Nets and ResNet are widely used in object detection and classification.

Girshick and his team have made important contributions to the task of image object detection and classification [5,6,7,8]. The region convolutional neural network (R-CNN) model [5] designed by Girshick was the first successful case of introducing deep learning into object detection. The spatial pyramid pooling (SPP-Net) designed by Kaiming He et al. [6] inputted the entire image into CNN, and the feature graph was obtained by the shared convolution calculation of all areas only once, and the test speed was 24–102 times faster than R-CNN in different tests data. Girshick [7] introduced the region of interest (RoI) pooling layer and multi-task loss function on the basis of SPP-Net, designed the Fast R-CNN model and realized the single-level training process. Shaoqing Ren et al. [8] designed breathtaking R-CNN based on Fast R-CNN using region proposal network (RPN), and the test time of a single figure is only 0.198 s. Jifeng Dai [9] changed the VGG16 in the Faster R-CNN to ResNet, further improving the accuracy.

When the above networks are applied to a traffic environment, the accuracy can be better improved by adjusting the network structure or scale distribution [10]. Ye Wang [11] optimized anchor generation and improved performance through region of interest allocation, making the number of features after pooling more suitable for final prediction. Yang Gao et al. [12] improved the convolution layer region scheme of the Faster R-CNN model and improved the detection accuracy by 7.3% through the KITTI data test. Reference [13] presents a pre-processing pipeline on Faster R-CNN to improve the training and detection speed. The pre-processing pipeline is based on the Sobel edge operator and Hough transform to detect lanes, and use a rectangular region to extract lane coordinates to reduce RoI.

Adverse weather has been endangering traffic safety. On the one hand, reduced visibility, slippery roads and other factors caused by adverse weather are one of the fundamental causes of traffic accidents. Shubhayu et al. [14] analyzed the fatal accident reporting system (FARS) data set prepared by the National Highway Traffic Safety Administration from 1994 to 2012, and the number of weather-related deaths during the study period accounted for about 16% of the total deaths. Reference [15] used the FARS data set from 2001 to 2012 to investigate the location and weather conditions of pile-up accidents in the United States. During the investigation period, more than 100 accidents resulted in 236 deaths, and the results showed that more than half of the accidents were related to weather conditions, especially reduced visibility. In the research of traffic safety in fog days, Anik Das [16] studied the difference in driving stability between foggy days and sunny days. It was found that the probability of lane deviation from standard deviation on foggy days was 1.37 times higher than that in normal conditions, but the visibility level on foggy days was not quantified. Ghasemzadeh [17] found that the lane deviation rate of drivers in rain was 2.2 times higher than that in sun. On the other hand, adverse weather also brings many negative effects on the detection effect of sensors in assisted driving. Reference [18] summarizes the influence principle of adverse weather on cameras, radars, and laser infrared radar (LiDAR). Kutila et al. [19] shows that the detection range of millimeter wave radar can be reduced by 45% under heavy rain. In addition, for targets in different radar cross sections (RCS), there are significant differences in rain backscattering effects. Reference [20] studies the influence on the detection effect of LiDAR under the condition of a stable fog environment in fog room. The wavelength of LiDAR is 905 nm (more than 90% LiDAR choose this wavelength), and the performance of LiDAR will be affected when the visibility range is reduced. In multiple tests, target detection performance was reduced by 50%.

In order to overcome the influence in adverse weather on assisted driving, there has been a focus on sensor support technologies under adverse weather. Lee Unghui [21] proposed a sensor fusion algorithm that can operate in a variety of weather conditions, including rainfall, combining LiDAR and camera information to detect the lane, but it is still difficult to use in the environment of fog or snow-covered road. Lisheng [22] presented a multi-class weather classification method to improve machine vision in bad weather situations based on multiple weather features and supervised learning. Reference [23] proposed a new architecture based on the Faster R-CNN model and convolutional neural network to restore the visibility of fuzzy images, which can achieve better target detection and image recovery in an adverse environment. In addition, some researches are devoted to the processing technology of foggy images [24,25,26] to enhance the accuracy of detection through image noise reduction technology.

Adverse weather brings great risks to traffic safety, and even advanced sensors to assist driving also do not perform well. As the most common sensor in assisted driving, the vision sensor is easily affected by visibility. Fog is a direct factor affecting visibility, it is necessary to verify its influence on vision sensor. However, none of the above studies involved the detection of a foggy day’s effect on visual sensors, nor did they quantify and grade visibility on foggy days, therefore, the accuracy of machine vision in foggy weather is always a fuzzy definition. Therefore, we hope to be able to research that in terms of visibility levels. On the basis of summarizing the deficiencies of previous studies, this paper takes the Faster R-CNN as an example to study the influence of foggy days on the detection effect of visual sensors, and quantifies the visibility level in the research process. This will lead to more precise research on weather and traffic safety, and also provide experiences for other research in this field.

## 2. Foggy Weather Environment Visual Imaging Model

### 2.1. Attenuation Model

Under foggy conditions, the scattering effect of incident light on suspended particles in the air will attenuate the light intensity that eventually enters the imaging equipment [27]. After a beam of parallel light passes through atmospheric space, the change in capability at *x* can be expressed as:

(1) $$\frac{dE\left(x,\lambda \right)}{E\left(x,\lambda \right)}=-\beta \left(\lambda \right)dx$$

where d is the distance between the target and the imaging device. Assume that the distance between the target scene and the imaging equipment is d, then integrate Equation (1) from 0 to d, and the result is that the light intensity received by the imaging equipment is:

(2) $$E\left(d,\lambda \right)=E_{0}\left(\lambda \right)e^{-\beta \left(\lambda \right)d}$$

where λ is the wavelength of incident light. $E_{0}\left(\lambda \right)$ is atmospheric scattering coefficient, represents the scattering ability of atmospheric light per unit volume. $\beta \left(\lambda \right)$ represents the actual intensity of the target obstacle light scattering through the atmosphere to the imaging equipment. From Equation (2), it is found that atmospheric attenuation has an exponential relationship with d.

### 2.2. Interference Model

Besides the attenuation of atmospheric scattering, the environmental imaging effect on foggy days is also affected by the surrounding light. The interference sources of ambient light include the interference of sunlight, sky and ground light, and reflected light in other scenes. The particles in the fog will scatter the ambient light, and the ambient light will change its propagation path under the scattering effect, and finally reach the imaging equipment. The interference imaging model [28] can be expressed as:

(3) $$E_{a}\left(d,\lambda \right)=E_{\infty }(\lambda )(1-e^{-\beta \left(\lambda \right)d})$$

where $E_{\infty }\left(\lambda \right)$ refers to the horizon brightness, and the interference degree of ambient light also increases with the target distance.

### 2.3. Foggy Weather Environment Visual Imaging Model

**Figure 1:**
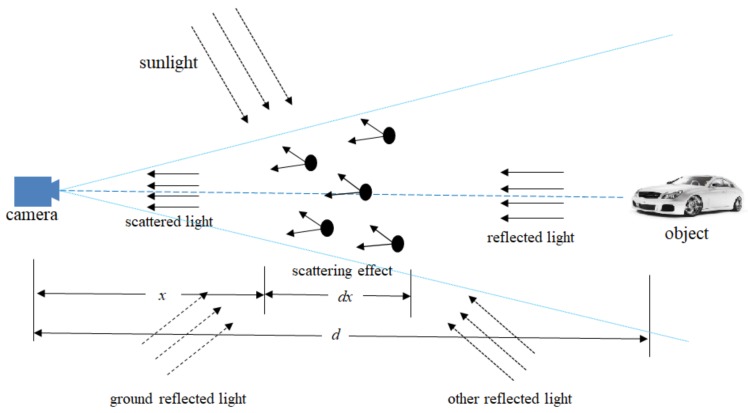
In foggy conditions, reflected light passes through attenuation and other interference before it reaches the imaging equipment from the target object, and the solid line shows atmospheric attenuation and the dotted line shows light interference.

A foggy weather environment visual imaging model can be seen as the result of superposition of the attenuation model and interference model [28], as shown in Figure 1. Affected by both actions, the quality of foggy images is seriously reduced. The mathematical model can be expressed as:

(4) $$E\left(d,\lambda \right)=E_{0}\left(\lambda \right)e^{-\beta \left(\lambda \right)}+E_{\infty }\left(\lambda \right)\left(1-e^{-\beta \left(\lambda \right)}\right)$$

The above model analyzes the optical principle of foggy image degradation from the perspective of physics. This model is also a foggy image model generally recognized in the field of visual imaging. The experimental simulation part of this study, the generation of foggy scenes and the acquisition of foggy images are all based on this model.

### 2.4. Feature Analysis of Foggy Images

**Figure 2:**
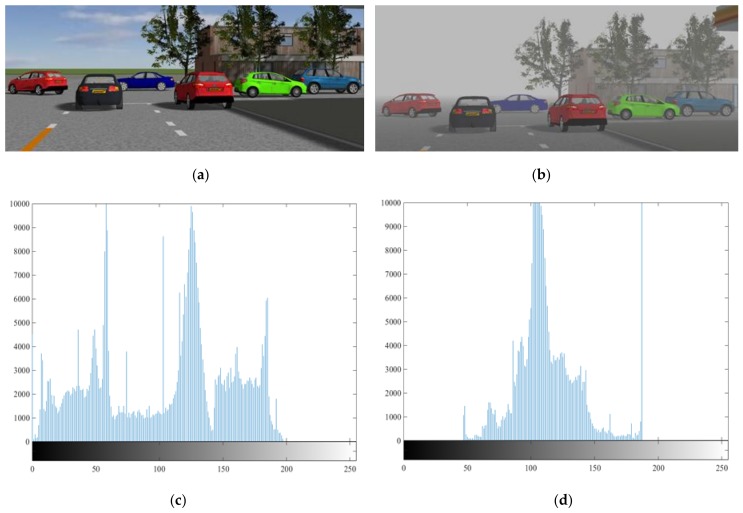
This is a contrast between the image of the weather and the grayscale of the foggy image, in which (**a**) is the image of sunny day, (**b**) is the image in visibility of 200 m of fog day, (**c**) is the grayscale of sunny day image, and (**d**) is the grayscale of fog day image.

The grayscale of the image can reflect the color information contained in the image, to a large extent, and also indirectly reflect the feature information. We can further analyze the influence of fog on imaging by comparing the grayscale between images in fog and without fog. As shown in Figure 2, based on the foggy day imaging model, we compared the grayscale of the sunny day image with the foggy day image in visibility of 200 m. It is obvious that under sunny conditions, the grayscale of sunny image is evenly distributed at 0–200, while, in the same scene, the grayscale of foggy day image is almost 0 at 0–50, and highly concentrated at 90–120. This reflects that the addition of fog effect changes the feature information of the original image, which could directly affect the image object detection effect.

In view of the above analysis, we found that obstacle detection of a visual sensor in fog has a certain research significance. In the subsequent experiments, we set up the experiment of machine vision obstacle detection under different visibility and different fog level according to the imaging contrast model by PreScan simulation software. Through experiments, we quantified the effect of fog on the detection effect of visual sensors to the level of visibility which is of great significance to the improvement of the obstacle detection method of machine vision in traffic scenes.

## 3. Obstacle Detection in Traffic Environment Based on Faster Region Convolutional Neural Network (R-CNN)

### 3.1. Building the Faster R-CNN Framework

**Figure 3:**
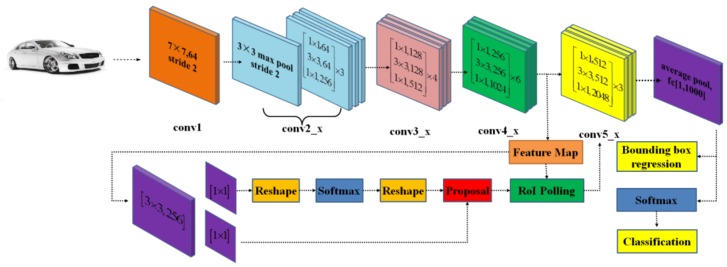
This is the construction of Faster R-CNN, and ResNet-50 is at the top of the figure to get feature map, and in the bottom of the figure is region proposal network (RPN), region of interest (RoI), and Softmax, respectively, from left to right.

In this paper we build the Faster R-CNN from four main parts: convolution layers, region proposal networks, RoI pooling, and classification. The framework of network construction is shown in Figure 3.

Compared with the initial network [8], the main functions and improvements of each part are as follows:

#### 3.1.1. Convolution Layers

Convolution layers are essentially a CNN-based target detection network. Image features are extracted by convolution, activation, pooling, and other multi-layer operations, which are used in the RPN layer and RoI layer. In order to improve the detection accuracy, ResNet-50 [4,9] was used for the CNN-basic network. In addition, the network connection structure was adjusted, and the output of feature map after conv4_x was used for sharing RPN and RoI. Conv5_x no longer extracts features, only converts features to 2028 dimensions through convolution, and finally uses average pooling for classification and frame regression.

#### 3.1.2. Region Proposal Networks (RPN)

Region Proposal Networks is used to generate regional proposals. After convolution layers, feature maps are divided into upper and lower layers after the ResNet convolution layer and convolution layer. The top layer is used for the Softmax classifiers’ background and ground truth and produces anchors, while the bottom layer is to calculate the bounding box regression offset of the anchors. Finally, the proposal layer integrates information from upper and lower layers, using anchors of ground truth and bounding box regression offsets to obtain an accurate proposal, and at the same time, removing any proposal that is too small or out of bounds. After the RPN networks, the objects have been located in the image.

#### 3.1.3. Region of Interest (RoI) Pooling

The function of RoI pooling layers is to unify the size of the feature maps. Firstly, we collect the information of feature maps and proposal layers, and convert the feature maps generated by images of different sizes into the same size, so as to facilitate the reception of the full connection layer and classifier behind. In order to reduce the loss of the original image caused by cutting or compression, max pooling should be done for each frame (16 × 16) of fixed size (4 × 4). Reference [29] proves that the effect of RoI alignment is better, so it is used to replace RoI pooling in the experiment.

#### 3.1.4. Classification

This is the last part of Faster R-CNN, in this section we will classify objects and calculate the final position of the bounding box. The location of ground truth object can be accurately calculated through bounding box regression, and the final classification can be completed by calculating proposal categories in the Softmax feature maps.

### 3.2. Loss Function

Loss function measures the difference between predicted value and real value. This paper follows the definition of multi-task loss in reference [7], and the loss function is expressed as in:

(5) $$L\left(\left\{p_{i}\right\},\left\{\left\{t_{i}\right\}\right\}\right)=\frac{1}{N_{cls}}\sum_{i}L_{cls}\left(p_{i},p_{i}^{*}\right)+\lambda \frac{1}{N_{reg}}\sum_{i}p_{i}^{*}L_{reg}\left(t_{i},t_{i}^{*}\right)$$

where the above equation is composed of two parts: Part 1 is classification loss and part 2 is bounding box regression loss, and $p_{i}$ is the probability that anchor predicts of the objects, and $p_{i}^{*}$ is the label of ground truth, $p_{i}^{*}=0$ for negative label and $p_{i}^{*}=0$ for positive label. The two parts are normalized by $N_{cls}$ and $N_{reg}$ and weighted by a balancing parameter λ.

#### 3.2.1. Classification Loss

From Equation (5) we know that the classification loss is expressed as:

(6) $$\frac{1}{N_{cls}}\sum_{i}L_{cls}\left(p_{i},p_{i}^{*}\right)$$

where $N_{cls}$ is the total number of anchors. The equation means calculated logarithmic loss for each anchor, and the sum is divided by the total number of anchors. We determined the value of $N_{cls}$ according to reference [8], which in the process of training RPN randomly chose 256 achors in an image to compute the loss function, and therefore, $N_{cls}$= 256, and in the training Fast R-CNN $N_{cls}$ = 128. where $L_{cls}\left(p_{i},p_{i}^{*}\right)$ is the logarithmic loss of object and non-objected, and it can be calculated as:

(7) $$L_{cls}\left(p_{i},p_{i}^{*}\right)=\log\left[p_{i}p_{i}^{*}+\left(1-p_{i}^{*}\right)\left(1-p_{i}\right)\right]$$

Equation (7) is a typical binary cross entropy loss.

#### 3.2.2. Bounding Box Regression Loss

From Equation (5) we know that the bounding box regression loss is expressed as:

(8) $$\lambda \frac{1}{N_{reg}}\sum_{i}p_{i}^{*}L_{reg}\left(t_{i},t_{i}^{*}\right)$$

where, $t_{i}$ representing the four parameterized coordinates of the predicted bounding box, and $t_{i}^{*}$ indicates the actual offset of anchor relative to ground truth label. $N_{reg}$ is the size of feature map, which is about 2400; the empirical value of *λ* is 10; $L_{reg}\left(t_{i},t_{i}^{*}\right)$ is calculated as:

(9) $$L_{reg}\left(t_{i},t_{i}^{*}\right)=R\left(t_{i}-t_{i}^{*}\right)$$

In the above equation, *R* is the $\mathrm{smooth}L_{1}$ function, as in Reference [7], it can be expressed as:

(10) $$\mathrm{smooth}L_{1}\left(x\right)=\left\{\begin{array}{ll} 0.5x^{2}\times \frac{1}{\sigma^{2}}, & \mathrm{if}\left|x\right|<\frac{1}{\sigma^{2}}\\ \left|x\right|-0.5, & \mathrm{otherwise} \end{array}\right.$$

## 4. The Effect of Fog Visibility on Detection Results

### 4.1. Data Preparation

**Figure 4:**
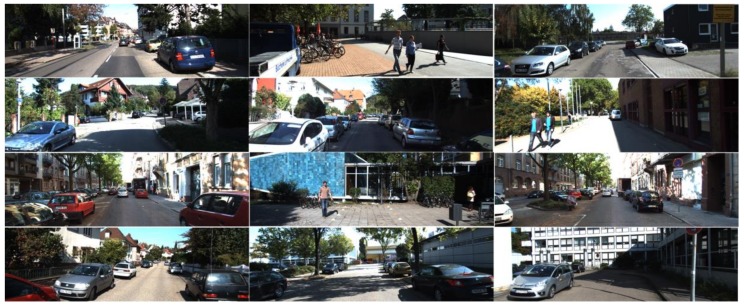
The KITTI data set, co-founded by the Karlsruhe institute of technology and the Toyota institute of technology, is the largest evaluation data set of computer vision algorithms in the world.

The main tasks of object detection of machine vision in the assisted driving field are detecting mostly vehicles and pedestrians, which requires training data to meet the special requirements of their missions. Therefore we choose KITTI [30] dataset to train the network, the data set contains real image data of urban, rural, and highway scenarios, and in each image as many as 15 cars and 30 pedestrians, and varying degrees of shade and truncation. KITTI data set is one of the most widely used data sets to study the superiority of machine vision algorithm in assisted driving. Some scenarios in the data set are shown in Figure 4.

The KITTI data set used in the experiment contains a total of 7841 pictures and data labels. We divide all pictures by proportion of train: Validata: Test = 8:1:1, and a total of 6732 pictures are used for training, 749 pictures are used for verification. In this experiment, to simplify the operation, the three categories of ‘Person_sitting’, ‘Motorists’ and ‘Pedestrian’ in the data label are unified into ‘Pedestrian’, while ‘DontCare’ and ‘Misc’ are excluded. After classification, the detection objects and their numbers are given in Table 1.

### 4.2. Experimental Process

We use Python programming and mixed of TensorFlow and Keras the Faster R-CNN. Firstly, we use ImageNet to initialize the model, and then to train RPN, and parameters are adjusted end to end. In the second step, we train the detection network, Fast R-CNN, and the proposals used to train come from RPN. Then the parameters of Fast R-CNN are used to adjust the RPN parameters. Finally, we fine-tune the remaining parameters of Fast R-CNN, using the proposals output of the adjusted RPN.

In the setting of network parameters we make the batch size = 64, and learning rate of RPN and CNN are to be determined. According to the above steps, we use the KITTI data set to train the network in chapter 3, and the experiment equipment include Intel core i5-8700 processor, NVIDIA GeForce GTX 1050 graphics card, and 8 G of memory.

**Figure 5:**
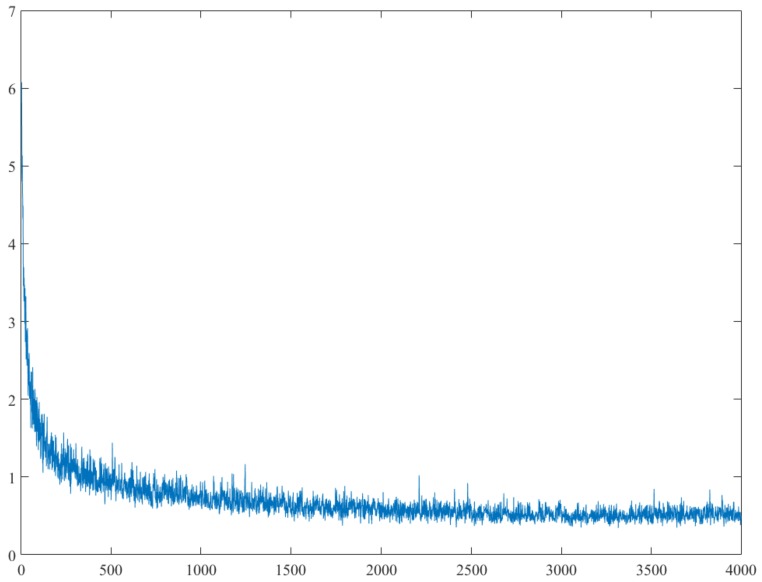
The abscissa represents the training times and the ordinate represents the total loss of the network, and the loss no longer shows a decreasing trend after training of 3000 times.

**Figure 6:**
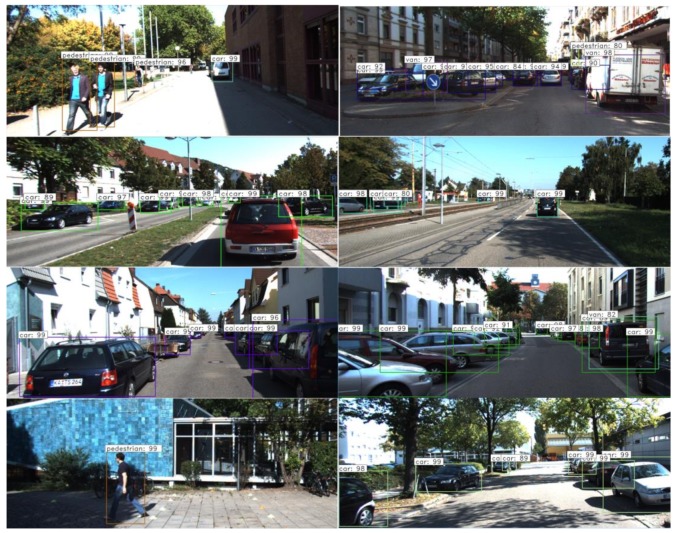
The picture shows the result that using the trained network to detect the test data set of KITTI.

Figure 5 shows the change of the loss function value in the training process, and when the change no longer shows a decreasing trend then stops train. Figure 6 shows the detection effect of the trained network on the source data set, in which the recall of detection is 93.83% (in object detection, recall measures the probability of ground truth objects being correctly detected [31]).

In a real environment, the weather factors were uncertain, so in order to make the study of visibility and fog levels more precise, we use the software to generate images of certain visible distance and divide them into different fog levels, and then we provide real environmental foggy images to verify the reliability of the simulation experiment.

**Figure 7:**
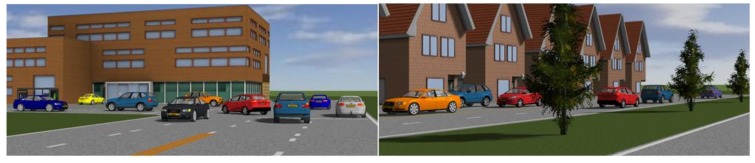
Part layout of the experiment scenes display, including model, colors, and shielding of cars.

In simulation, the fog environment is built by the PreScan software based on the fog imaging model. PreScan is a physics-based simulation platform based on sensor technologies such as radar, camera, and GPS. The software contains people, vehicles, roads, buildings, trees, and other objects, so we can set up many kinds of traffic scenes and set cameras on our test vehicle to obtain pictures of the scenes. In addition, weather conditions can also be set in PreScan, especially the visibility distance in fog, so that our study on the effectiveness of machine vision in fog can reach the level of visibility. We set up different traffic scenes, and obtained the scene images by installing the camera on the test vehicle, as in shown Figure 7.

Among the labels of KITTI data set, the most common object is vehicle included nearly 30,000, followed by less than 5000 pedestrians. In addition, in the real driving environment vehicles represent more driving obstacles. Therefore, considering of the sufficient sample size and real environment, we use vehicle detection object.

A total of 20 traffic scenes were set up in the simulation experiment, each of which contained 5 to 14 target vehicles. The diversity of vehicles were reflected by vehicle type, body color, and shielding. The vehicle type included 8 models, which are Audi A8, BMW X5, Toyota Previa, etc. In addition, the color of the vehicle body was randomly set. In the experiment, the vehicle was shielded to different degrees by trees, buildings, and other vehicles. All those were to further enrich the diversity of the objects to be tested.

According to the classification standards of fog level and visibility, we divide foggy weather into six conditions according to visibility, as shown in Table 2. Among them, the detection result of clear (no fog) and misty weather is almost the same, while the detection effect is extremely poor in dense fog, so these two weather conditions are not studied in this paper. For the other four weather conditions, we set specific visibility for each traffic scene in the experiment. Due to the large span visibility between the two levels of slight fog and heavy fog, we set two kinds of visibility at 500 m and 300 m in moderate foggy weather. Therefore, we set 5 visibility levels for each scene in this paper, respectively are less than 200 m, 200~300 m, 300~500 m, 500 m, 800 m, and more than 800 m, as shown in Table 2. After setting up the experimental scene, the recognition effect can be detected through the trained Faster R-CNN network.

### 4.3. Results

**Figure 8:**
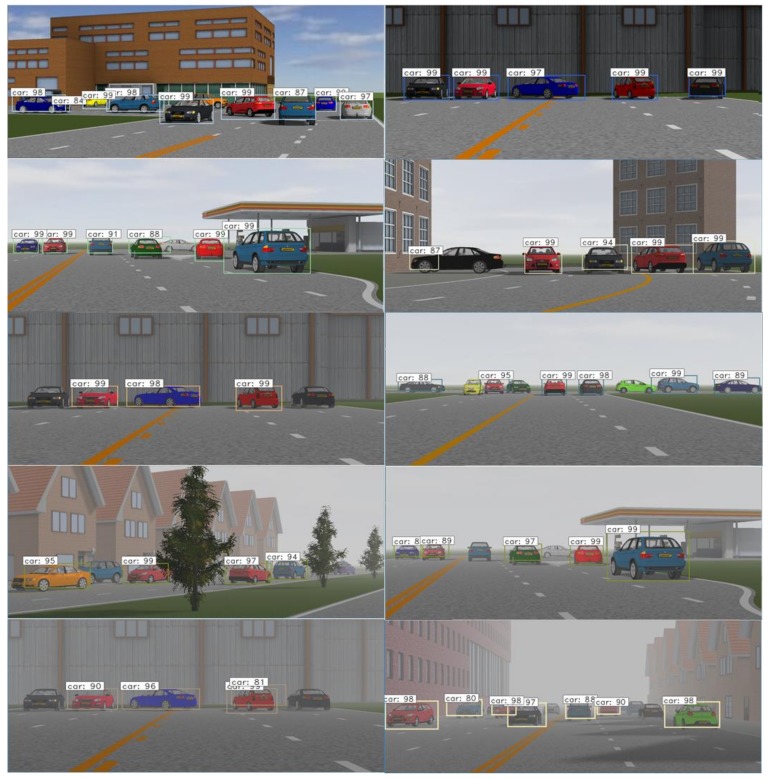
Display of the same scene in different visibility level, and the visible distance in picture with five layers from top to bottom are without fog, 800 m, 500 m, 300 m, and 200 m.

The detection result under various weather conditions is shown in Figure 8. It can be seen that in Figure 8 from top to bottom, with the visibility declining in foggy days, the objects to be detected in the scene gradually become blurred, and the network’s ability to identify the objects decreases. In addition, by comparing the color of the cars and recognition result in Figure 8, it can be found that the ability of machine vision on vehicle objects’ detection in foggy days is also related to vehicle colors. We can see that the ability of machine vision has the worst recognition on a black vehicle, while red is less affected by foggy days. The explanation of this phenomenon can be traced back to the foggy day imaging model. Different colors of light have different wavelengths, among which red has a longer wavelength. During the foggy day imaging process, vehicle with red color get the minimum suffer by attenuation and scattering in a foggy environment, so it can keep more characteristic information and can be detected more easily.

The decrease of visibility on foggy days directly leads to the decline of detection accuracy, but when the visible distance is over than 800 m, it has little impact on the detection result. When fog level is strengthened and visibility is less than 800 m, the detection accuracy decreases significantly. The accuracy of recognition in moderate fog is about 65%~73%, and is lower than 57.75% in heavy fog. The results are shown in Table 3. At this time, obstacle detection technology based on machine vision has been unable to meet the needs of assisted driving safety, and the confidence of visual sensor information in assisted driving needs to be adjusted reasonably.

### 4.4. Verification in Real Scene

The above detection results at different distances are obtained through simulation experiments based on the foggy day imaging model, so the simulation results need to be further verified. The reliability of the above conclusion can be judged by comparing the results of a real scene and simulation experiment. However, as mentioned before, the randomness of weather factors makes it impossible for us to obtain the fog environment at each visible distance or level, so we cannot verify the above results in each visible distance, and they can only be proved indirectly by verifying partial results.

**Figure 9:**
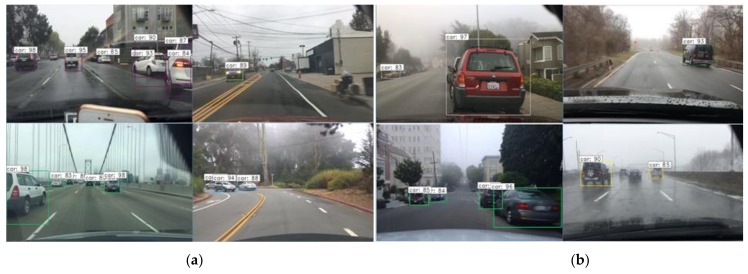
Display of the real scene detection results (**a**) in moderate fog, (**b**) in heavy fog.

We use the BDD100K [32] data set to verify the simulation results. The BDD100K data set, provided by the artificial intelligence (AI) laboratory of Berkeley University, is the largest and most diverse open driving data set at present. Compared with the KITTI data set, the BDD100K contains driving scenes under different weather conditions and marks objects in the scene in detail [33]. It is also crowd-sourced, and covers a very large area and diverse visual phenomena, but it is very clearly limited to monocular RGB image data from cameras on vehicles. We can find out the driving scene in foggy weather from the data set and obtain its label information. In the picture, the visibility in fog is difficult to quantify, so we only detect the real scene under medium fog and heavy fog. The detection results are shown in Figure 9.

Table 4 shows the detection results under real scenarios, among which the accuracy is 68.02% in medium foggy weather and 58.41% in heavy fog. By comparing the accuracy of each fog weather level in Table 3, it can be seen that the detection recall in real scenes corresponds to the accuracy interval given under experimental conditions. The detection experiment in real scenes further verifies the influence of foggy weather on visual sensor and verifies the validity of the simulation experiment.

## 5. Conclusions

This study is based on the increasingly mature machine vision technology, the purpose of which is to analyze the influence of weather factors on its detection accuracy. Firstly, we discussed the characteristics of visual imaging in a foggy environment and its influence on object detection theoretically. Then we built Faster R-CNN as the basic network and training with KITTI data set. Based on a foggy day imaging model, we used PreScan software to generate 4 weather conditions: sunny (no fog), light fog, medium fog and heavy fog, and obtained detection results of them. Furthermore, we used real scenes under moderate fog and heavy fog conditions to verify that the results from simulation are reliable. The detection recall of the foggy environment is 91.55% in sunny, 85.21% in light fog, 72.54%~64.79% in moderate fog, and less than 57.75% in heavy fog. Considering the sufficient sample size and real environment, we used vehicles as detection objects. In future work, we will enrich the sample size of pedestrian and include it in the detection object.

With the application of advanced sensors, many traditional traffic problems have been solved, but the challenges caused by adverse weather still cannot be avoided. In this paper, we combined the foggy environment with machine vision, and quantified the effect of fog on machine vision. Through this study, we can determine the impact of bad weather on the detection results of assisted driving, so that we can improve the safety of assisted driving through further research.

From the analysis of the grayscale characteristics of the foggy image, we can see that the color information of the foggy image changes a lot, which will also lead to the change of the feature information in the image, and the detection networks trained by sunny day environment images could not recognize these changes well. Therefore, in the next research work, we can add objects to foggy images to the training set so as to make the machine vision contain these foggy image features and improve detection accuracy.

Assisted driving decisions need to obtain information from sensors. Therefore, the detection accuracy of sensors can directly affect the confidence level of the information. In this paper, we give the detection recall under different fog levels, which can help to determine confidence in visual sensor information at different levels. Furthermore, other sensors will also be affected by bad weather, and this article can provide the experimental methods for the other issues under adverse weather. Also, the existing sensor information fusion algorithm has largely failed to consider the weather condition, so in the next step of research we will study the information fusion algorithm considering the effects of the weather.

Of course, the experiment in this paper has some shortcomings. There are some differences among various obstacle detection algorithms, but the overall difference is not obvious. The Faster R-CNN selected in this paper is roughly the same as other algorithms in terms of methods and principles, which were broadly representative, so this paper can fully reflect the problems we studied.

## Figures and Tables

**Table 1:** Types and quantities of objects for Faster R-CNN training.

Classes	Car	Pedestrian	Tram	Truck	Van	Background
**Number of classes**	28,742	4709	511	1094	2914	—

**Table 2:** Fog level and visible distance set in the experiment.

Weather	Clear	Mist	Slight Fog	Moderate Fog		Heavy Fog	Dense Fog
**Visual distance (km)**	>10	10~1	<1	0.5~0.2		0.2~0.05	<0.05
**Experiment value (m)**	—	—	800	500	300	200	—

**Table 3:** Detection recall of Faster R-CNN in different visual distance.

Fog Level	Clear	Slight Fog	Moderate Fog		Heavy Fog
**Visual distance (m)**	—	800	500	300	200
**Detection recall (%)**	91.55	85.21	72.54	64.79	57.75

**Table 4:** Object detection recall in real fog environment.

	Real Sense Number	Object Number	Successful Detection Number	Recall
**Moderate fog**	50	172	117	68.02%
**Heavy fog**	50	113	66	58.41%
